# Identification and Validation of Prognostic Biomarkers Specifically Expressed in Macrophage in IgA Nephropathy Patients Based on Integrated Bioinformatics Analyses

**DOI:** 10.3389/fmolb.2022.884588

**Published:** 2022-05-05

**Authors:** Yuqing Ding, Hua Li, Lichen Xu, Yukun Wang, Huiying Yang

**Affiliations:** ^1^ Department of Nephrology, Sir Run Run Shaw Hospital, Zhejiang University School of Medicine, Hangzhou, China; ^2^ Department of Urology, Sir Run Run Shaw Hospital, Zhejiang University School of Medicine, Hangzhou, China

**Keywords:** IgA nephropathy, single-cell RNA sequencing, bulk transcriptome, integrated bioinformatics analyses, prognostic biomarkers

## Abstract

**Background:** Immunoglobulin A nephropathy (IgAN) is the most common type of primary glomerulonephritis worldwide and a frequent cause of end-stage renal disease. The inflammation cascade due to the infiltration and activation of immune cells in glomeruli plays an essential role in the progression of IgAN. In this study, we aimed to identify hub genes involved in immune infiltration and explore potential prognostic biomarkers and therapeutic targets in IgAN.

**Methods:** We combined the single-cell and bulk transcriptome profiles of IgAN patients and controls with clinical data. Through single-cell analysis and weighted gene co-expression network analysis (WGCNA), Gene Ontology (GO) enrichment analysis, and differentially expressed gene (DEG) analysis in the bulk profile, we identified cell-type-specific potential hub genes in IgAN. Real hub genes were extracted via validation analysis and clinical significance analysis of the correlation between the expression levels of genes and the estimated glomerular filtration rate (eGFR) in the external dataset. Gene set enrichment analysis was performed to predict the probable roles of the real hub genes in IgAN.

**Results:** A total of eleven cell clusters were classified via single-cell analysis, among which macrophages showed a variable proportion between the IgAN and normal control samples. We recognized six functional co-expression gene modules through WGCNA, among which the black module was deemed an IgAN-related and immune-involving module via GO enrichment analysis. DEG analysis identified 45 potential hub genes from genes enriched in GO terms. A total of twenty-three potential hub genes were specifically expressed in macrophages. Furthermore, we validated the differential expression of the 23 potential hub genes in the external dataset and identified nine genes with prognostic significance as real hub genes, viz., CSF1R, CYBB, FPR3, GPR65, HCLS1, IL10RA, PLA2G7, TYROBP, and VSIG4. The real hub gens are thought to contribute to immune cell regulation, immunoreaction, and regulation of oxidative stress, cell proliferation, and material metabolism.

**Conclusion:** In this study, we demonstrated that macrophages infiltrated the glomeruli and contributed to the inflammatory response in IgAN. Based on integrated bioinformatics analyses of single-cell and bulk transcriptome data, we highlighted nine genes as novel prognostic biomarkers, which may enable the development of innovative prognostic and therapeutic strategies for IgAN.

## Introduction

Immunoglobulin A nephropathy (IgAN) is the most common primary glomerulonephritis worldwide (McGrogan et al., 2011; Kidney Disease, 2021) and is characterized by histopathological criteria of mesangial IgA deposits on renal biopsy. The clinical features of IgAN are highly variable, ranging from isolated microscopic hematuria to massive proteinuria and subsequent progressive renal failure. Approximately, 25–30% of IgAN patients develop end-stage renal disease within 20–25 years (Wakasugi, 2014; Trimarchi et al., 2017). Current pharmacotherapy for IgAN mainly focuses on renin–angiotensin system inhibitors, glucocorticoids, and immunosuppressants (Magistroni et al., 2015), which means that there are limited efficient and specific therapies. Thus, further studies are required to elucidate the pathogenesis of IgAN to discover novel therapeutic approaches for this disease.

IgAN is generally recognized as an autoimmune disease. The widely accepted “multi-hit” hypothesis was proposed to explain the major pathogenesis of IgAN (Perše and Veceric-Haler, 2019). The first hit is the overproduction of galactose-deficient IgA1 (Gd-IgA1) by B cells, followed by the recognition of Gd-IgA1 by specific IgG autoantibodies, with subsequent formation of circulating immune complexes and glomerular deposition as the next two hits. Persistent immune complex deposition triggers mesangial proliferation and inflammatory responses, eventually leading to glomerular injury (Suzuki et al., 2011).

Nevertheless, glomerular deposition of Gd-IgA1–IgG alone is insufficient to fully explain the pattern of IgAN. Multiple studies have indicated that immune dysfunction and immune cell infiltration also play a significant role in the development of IgAN, especially from mesangial deposition to renal injury (Silva et al., 2012; Wang and Harris, 2019; Takahata et al., 2020). Immune cell infiltration has been observed in both IgAN murine models and the kidneys of IgAN patients, and it is associated with a poor clinical outcome. These studies have confirmed the importance of immune cell infiltration and activation in IgAN. However, the expression characteristics of immune cells and regulatory mechanisms of immune infiltration in IgAN remain unclear. Therefore, exploring the molecular mechanisms of immune cells in the progression of IgAN is expected to enable the discovery of key elements with therapeutic potential.

Advances in high-throughput next-generation sequencing technology and bioinformatics have provided researchers with new avenues for elucidating the mechanisms underlying diseases. In particular, the rapid development of single-cell RNA sequencing (scRNA-seq) technology in recent years offers researchers a new alternative for studying the molecular expression characteristics of different cell atlases in diseases at single-cell resolution. However, there are some disadvantages to both bulk transcriptomic and scRNA-seq analyses. Bulk transcriptomic analysis could provide new clues to the relationships between expression patterns of genes and clinical characteristics but locating the expression of the genes in tissues is difficult. However, scRNA sequencing is usually conducted with a relatively small sample size, which reduces the reliability of the studies and partly limits clinical transformation. Therefore, integrative bioinformatics analyses of scRNA-seq and bulk transcriptomes are required to uncover the pathogenesis of IgAN.

In the present study, we performed integrated bioinformatics analyses combining single-cell and bulk transcriptomic data of renal tissues of IgAN samples for the first time, aiming to explore gene involvement in the activation of immune cells and to extract potential prognostic markers and precise targets specifically expressed in immune cells.

## Materials and Methods

### Study Design

**FIGURE 1:**
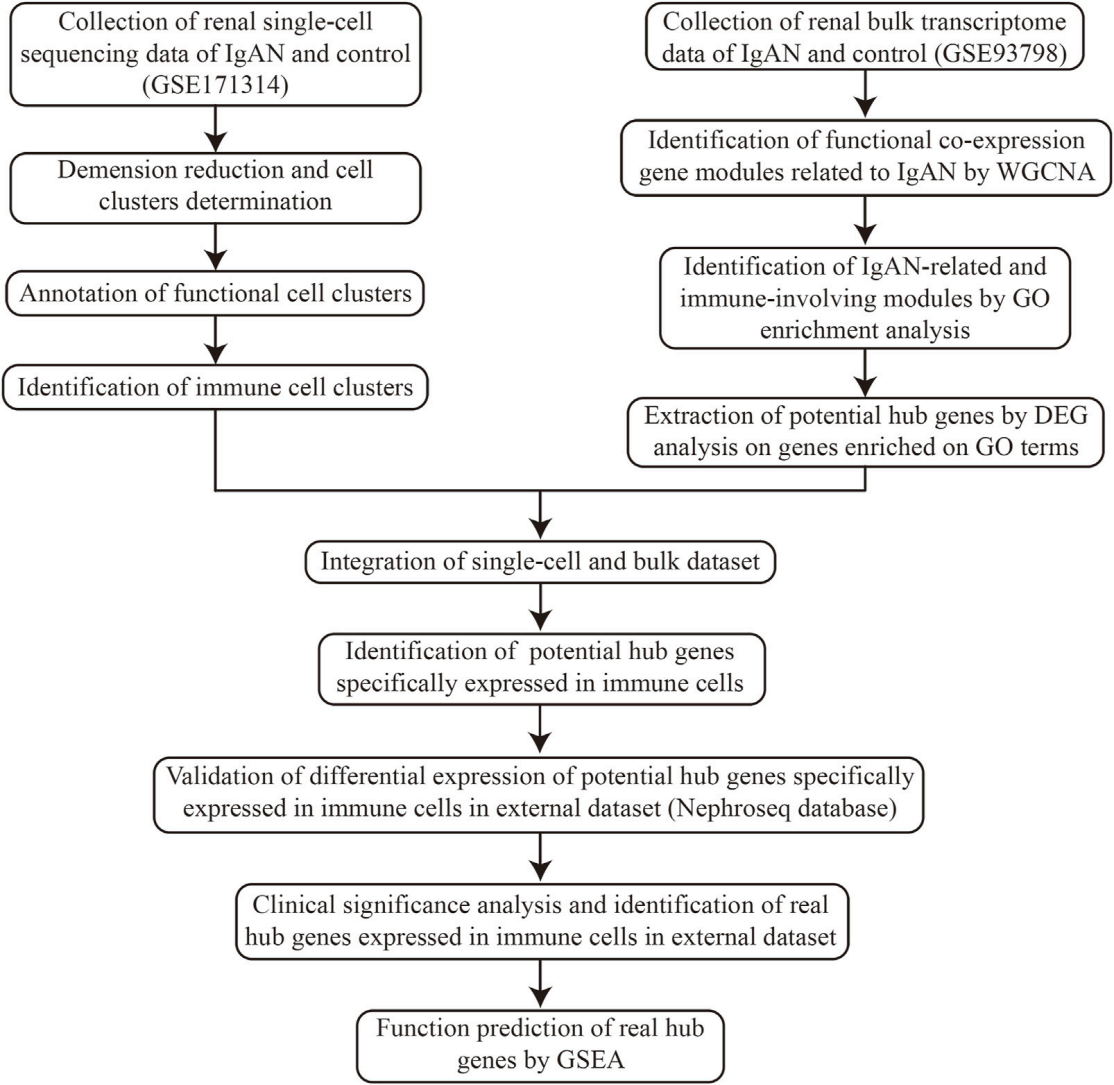
Workflow of the design and overall procedures of this study.

The overall design and workflow of the study are abstracted in Figure 1.

### Data Collection

We obtained the scRNA sequencing dataset (GSE171314) of renal tissues from four IgAN patients and one control from the Gene Expression Omnibus (GEO) database. The bulk gene expression data (GSE93798) by microarray of glomerular tissues from 20 IgAN patients and 22 healthy living donors were collected from the GEO database. The expression profiles of glomerular tissues with clinical traits from 27 patients with IgAN and 21 living donors were obtained from the Nephroseq database, which was regarded as an external dataset.

### Data Process of the scRNA Profile and Identification and Annotation of Cell Clusters

The scRNA sequencing data of GSE171314 contained 20,570 cells (6,438 cells from one healthy living donor and 14,132 cells from four IgAN patients). All analyses of the scRNA profiles were conducted following the standard workflow provided by the Seurat package (version 4.1.0, https://satijalab.org/seurat/) in R (version 4.1.2).

First, 2000 highly variable genes were identified, respectively, for the five scRNA profiles. Cells from the five samples were anchored to eliminate the batch effect (Stuart et al., 2019).

Subsequently, the expression values of all genes were normalized and scaled to ensure the data comparability. Principal component analysis was conducted, and the appropriate principal components for dimension reduction were decided based on ElbowPlot analysis. Uniform Manifold Approximation and Projection (UMAP) was applied to classify cells into different cell clusters at a proper resolution.

Cell cluster annotation was conducted based on the known biomarkers of different cells in renal tissues published in earlier studies (Table 1) (Suzuki et al., 2011; Wakasugi, 2014; Ohashi et al., 2016; Young et al., 2018; Liao et al., 2020; Chen et al., 2021; Li et al., 2021). Differentially expressed genes (DEGs) among different clusters were identified to validate the reasonableness of cell cluster annotation.

**TABLE 1:** Gene markers used for the annotation of cell clusters.

Cell type	Gene marker
Proximal tubule	CUBN, SLC13A1, LRP2, and ALDOB
Loop of Henle	SLC12A1 and CLDN16
Distal tubule	SLC12A3, CALB1, and SLC8A1
Principal cells	AQP2 and AQP3
Intercalated cells	SLC26A7, SLC4A1, and AQP6
Mesangial cells	FHL2, CTGF, MYL9, and ACTN1
Podocyte	NPHS2, PODXL, PTPRO, and PCOLCE2
Smooth muscle cells	ACTA2, TAGLN, MYH11, and MYLK
Endothelial cells	PECAM1, KDR, FLT1, and PLVAP
Macrophages	PTPRC, LYZ, CD68, and C1QA

### Data Preprocessing and Weighted Gene Co-Expression Network Analysis of Bulk Expression Profile

To identify key molecules specifically expressed in immune cells in IgAN by analyzing single-cell profiles, we first attempted to identify potential hub genes involved in immunoreaction by analyzing the bulk data with weighted gene co-expression network analysis (WGCNA) (Langfelder and Horvath, 2008).

The data preprocessing procedure was as follows: probe annotation was conducted for the GSE93798 dataset using the microarray platform file GPL22945 to convert the expression matrix of the probe into that of the official gene symbol. To eliminate the background noise produced by the non-varying genes, we retained genes with a top 5000 standard deviation in the expression matrix for WGCNA. Before performing regular WGCNA, we applied sample clustering to exclude possible outlier samples.

The WGCNA was conducted using the WGCNA R package (version 1.70-3). The expression matrix was transformed into Pearson’s correlation matrix using Pearson’s correlation analysis. According to an appropriate *β*-value (soft-thresholding value) with a relatively high scale-fit index and mean connectivity, the correlation matrix was transformed into a scale-free network after power operation. Then, considering indirect correlations, the scale-free network was converted into a topological overlap matrix (TOM). Genes were then divided into co-expression modules after applying average linkage hierarchical clustering based on the TOM-based dissimilarity measure.

To identify IgAN-related co-expression modules, we conducted module–trait correlation analysis by calculating Spearman’s correlation coefficient between clinical traits and module eigengenes of each module.

### Identification of Potential Hub Genes Related to Immunoreaction in IgAN by Enrichment Analysis and Differentially Expressed Gene Analysis

After identifying the IgAN-related co-expression modules, we performed enrichment analysis to clarify the biological functions of the modules and recognize immune-related modules. Gene Ontology (GO) enrichment (Lu et al., 2008) was conducted on genes clustered into certain modules using the clusterProfiler R package (version 4.2.2) (Yu et al., 2012). GO terms with a *p*-value less than 0.001 and Benjamin–Hochberg-adjusted *p*-value less than 0.01 were deemed as significantly enriched GO terms. Modules with enriched immune-related GO terms are considered to play a key role in immune disorders during the onset and progression of IgAN.

DEG analysis was performed between IgAN and normal controls for genes enriched in immune-related GO terms using limma R package (Ritchie et al., 2015). Genes with a *p*-value < 0.01, Benjamin–Hochberg-adjusted *p*-value < 0.05, and |log2fold change (logFC)| > 1 were considered DEGs. DEGs enriched in immune-related GO terms were regarded as potential hub genes, the expression locations of which should be defined in a single-cell profile.

### Identification of Cell-Type-Specific Potential Hub Genes in the scRNA Profile

The differential expression of certain genes in the bulk RNA profile represents their overall expression levels in the glomerular tissues, but one could not clarify the cell clusters that contributed to the significant upregulation or downregulation.

To investigate whether the potential hub genes were specifically expressed in the given cell clusters, we explored the expression level of these genes among different cell clusters in the scRNA profile and determined cell-type-specific genes. These genes were defined as cell-type-specific potential (CTSP) hub genes in IgAN.

Clinical Significance Analysis for Cell-Type-Specific Potential Hub Genes and Identification of Real Cell-Type-Specific Hub Genes in the External Dataset

To enhance the reliability of the CTSP hub genes, we first validated their differential expression between IgAN and the normal control in an external dataset (Ju CKD Glom from the Nephroseq database). The criteria for DEGs were set at *p* < 0.01, Benjamin–Hochberg-adjusted *p*-value < 0.05, and |log2fold change (logFC)| > 0.5.

Because the estimated glomerular filtration rate (eGFR) is a direct prognostic factor for IgAN patients, we analyzed the correlation between CTSP hub genes and eGFR in IgAN patients in the external dataset. The criterion for significant correlation was set at *p* < 0.05. Genes significantly correlated with eGFR were ultimately deemed real hub genes that play vital roles in the pathogenesis of IgAN and are expressed specifically in certain cell clusters.

### Function Prediction of Real Hub Genes via Single-Gene Gene Set Enrichment Analysis

Single-gene gene set enrichment analysis (GSEA) (Subramanian et al., 2005) was conducted for the real hub genes to explore their functions in IgAN. The expression profiles of GSE93798 were divided into high- and low-expression groups based on the expression levels of the real hub genes. Significantly enriched GO terms in the high-expression groups were identified following the criteria of *p* < 0.01 and false discovery rates (*FDRs*) < 0.25.

## Results

### Identification and Characterization of Cell Clusters in the scRNA Profile of Renal Tissues From IgAN and Normal Controls

**FIGURE 2:**
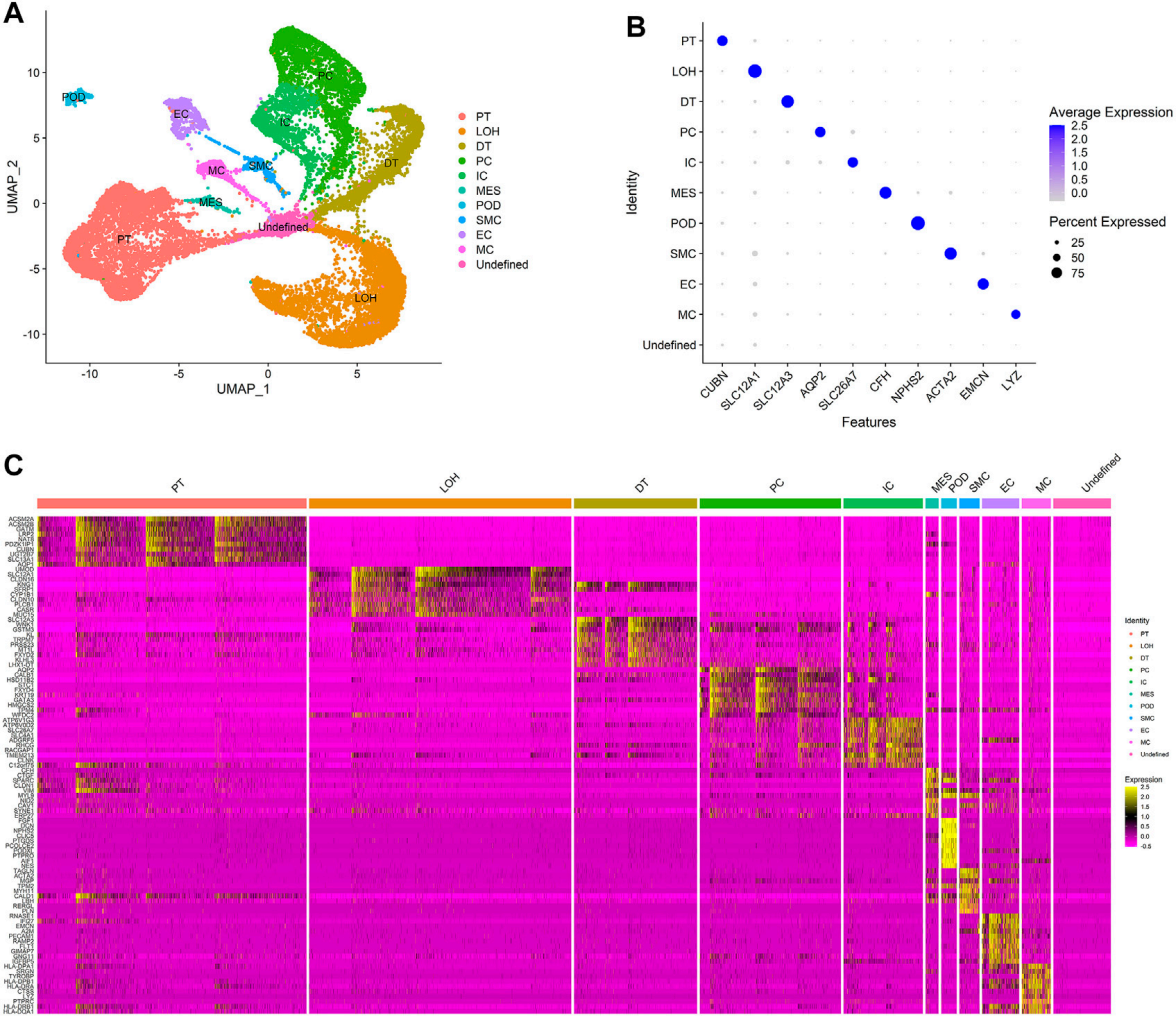
Cell clusters in kidneys of normal control and IgAN delineated by single-cell transcriptomic analysis. **(A)** UMAP plots of integrated profiles of renal cells colored by cell type. PT, proximal tubule; LOH, loop of Henle; DT, distal tubule; PC, principal cells; ICA, intercalated cells; MES, mesangial cells; POD, podocyte; SMC, smooth muscle cells; EC, endothelial cells; MC, macrophages. **(B)** Dot plots of the expression level of representative marker genes across 11 cell clusters. The size of the dot represents the proportion of the cell population that expresses the gene. Shading of color indicates the expression level of the gene. **(C)** Heatmap of the expression levels of top 10 marker genes in each cell cluster.

Dimension reduction was conducted with 15 principal components based on the results of ElbowPlot analysis (Supplementary Figure S1), and the cells were classified into 11 clusters based on the UMAP analysis at a resolution of 0.2. Cell clusters were annotated with known markers, as shown in Figure 2A and Figure 2B. The 11 clusters included endothelial cells (EC), mesangial cells (MES), podocytes (POD), proximal tubular cells (PT), distal tubular cells (DT), loop of Henle cells (LOH), principal cells (PC), intercalated cells (IC), macrophages (MC), smooth muscle cells (SMC), and an undefined cluster. The markers used for the cell cluster annotation are listed in Table 1.

In this study, we mainly focus on the effect of immune cell infiltration on IgAN. The results of cell clustering showed that the macrophages were the main immune cells that infiltrate the renal tissues of IgAN.

Except for known markers from previous studies, DEG analysis among clusters identified more marker genes that were specifically expressed in different clusters (Figure 2C), revealing that the annotation of the cells was accurate and made biological sense.

**FIGURE 3:**
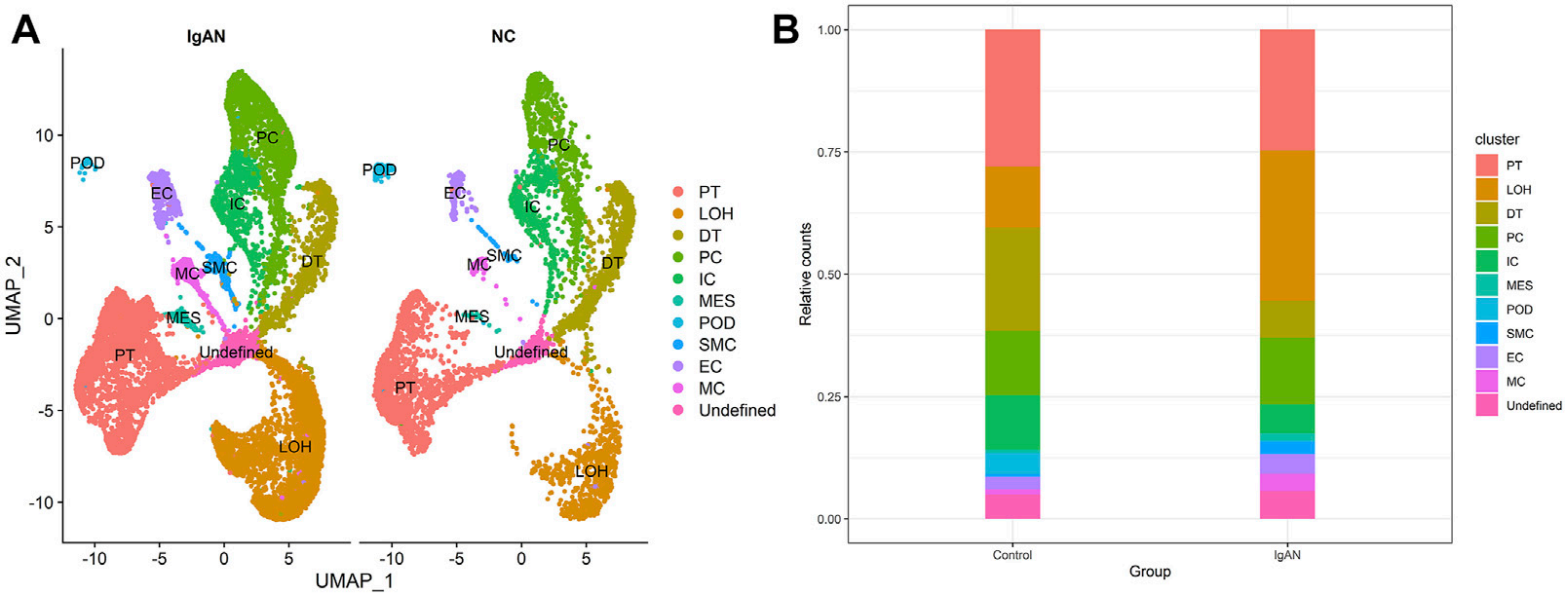
Single-cell landscape of renal cells in IgAN and NC groups. **(A)** UMAP plots of IgAN and NC groups. **(B)** Bar chart visualization of cluster distribution in IgAN and NC groups.

Figure 3 shows the distribution of cell clusters in IgAN and normal controls. As shown in Figure 3B, the proportion of LOH increased in IgAN samples, and that of DT, IC, and MES decreased in IgAN. Remarkably, although the quantity of immune cells that infiltrated the renal tissues is extremely low compared with renal parenchymal cells, the proportion of macrophages in IgAN increased by two times (from 1 to 3%) compared to that of normal controls. Hence, the infiltration and activation of macrophages in renal tissue might play essential roles in the pathogenesis of IgAN.

### WGCNA and Identification of the IgAN-Related Co-Expression Module in the Bulk Expression Profile

Sample clustering detected no outlier samples in the GSE93798 dataset (Supplementary Figure S2). As IgAN samples and normal controls were clustered together, all samples were retained for WGCNA.

**FIGURE 4:**
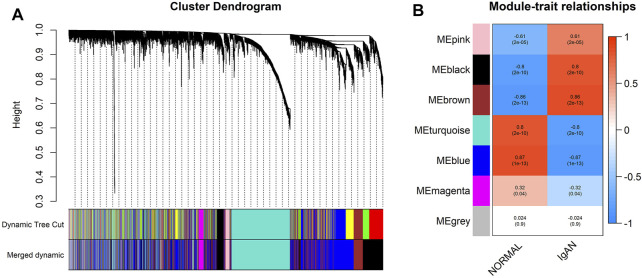
Division and identification of the co-expression network. **(A)** Cluster dendrogram of all genes based on a dissimilarity measure. The modules labeled by color are displayed at the bottom. **(B)** Heatmap of module–trait correlations. Each column contains the correlation coefficients and statistical significance. The black and brown modules were identified as IgAN-related modules.

The expression matrix was transformed into a TOM following the procedures mentioned previously. Seven was selected as the appropriate *β*-value for the construction of the scale-free network because it provided a relatively higher mean connectivity when the scale-fit index reached 0.85 (Supplementary Figure S3). All 5,000 genes were divided into six co-expression modules, and the gray module contained all genes that could not be divided into co-expression modules (Figure 4A). Supplementary Table S1 lists information on modules and their genes.

Through module–trait correlation analysis, we identified two IgAN-related co-expression modules (black and brown, Figure 4B).

### Identification of IgAN-Related Modules Involved in Immunoreaction

GO enrichment analysis was conducted on IgAN-related modules to investigate the biological functions of the genes in these co-expression modules.

**FIGURE 5:**
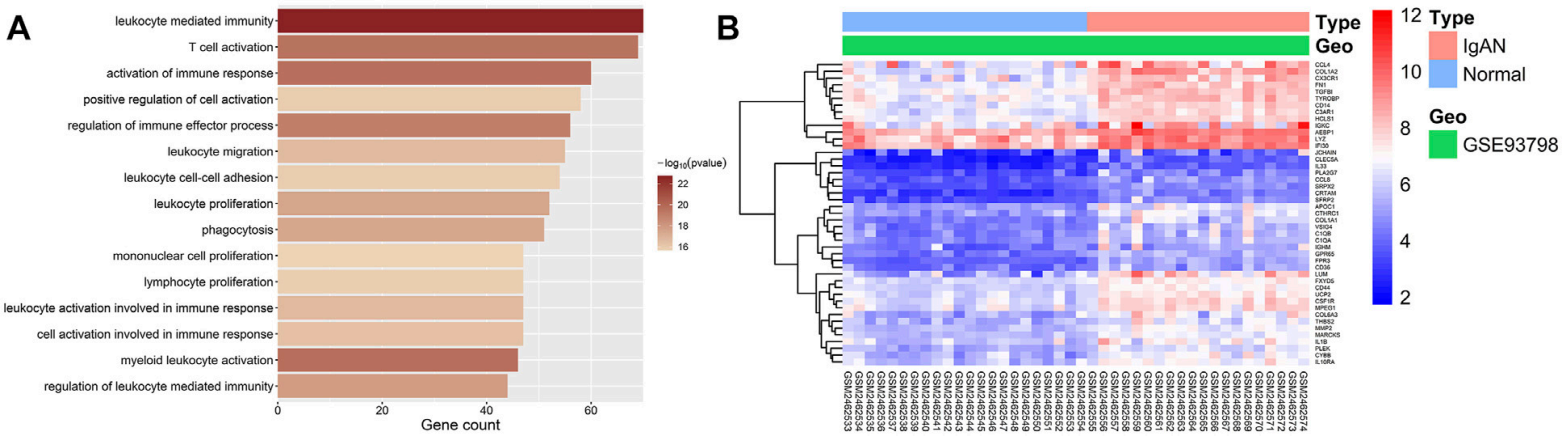
GO enrichment and DEG analysis of two trait-related modules. **(A)** Bar graphs showing the top 15 significantly enriched GO biological process (BP) terms in the IgAN-related and immune-involving module (black module). The shading of color represents the enrichment significance of each term, and the *x*-axis indicates the enriched gene count. **(B)** Heatmap of DEGs enriched in GO-terms in the IgAN-related and immune-involving module. The color of each cell represents the expression level of a gene in a sample (red for high level and blue for low level).

The biological significance of genes in the black module was mainly related to the activation, proliferation, and migration of immune cells as well as the activation of immune response (Figure 5A exhibits the top 15 significantly enriched GO terms), for example, “leukocyte-mediated immunity” (enriched gene count = 70, *p* = 1.76E-23), “activation of immune response” (enriched gene count = 60, *p* = 2.22E-20), “T-cell activation” (enriched gene count = 69, *p* = 2.94E-20), “leukocyte proliferation” (enriched gene count = 52, *p* = 3.16E-18), and “leukocyte migration” (enriched gene count = 55, *p* = 2.41E-17). Therefore, the black module was defined as an IgAN-related and immune-involving module for subsequent hub-gene mining. All GO enrichment results are presented in Supplementary Table S2.

### Screening of Potential Hub Genes via DEG Analysis

DEG analyses were conducted on genes enriched in GO terms in the IgAN-related and immune-involving (black) module. All 45 DEGs in this module were upregulated. The DEGs enriched in GO terms were regarded as potential hub genes for subsequent studies. Figure 5B shows a heatmap of all DEGs in the IgAN-related and immune-involving module. Details on all DEGs are available in Supplementary Table S3.

### Identification of Cell-Type-Specific Potential Hub Genes Expressed in Macrophages in the Single-Cell Profile

As the differential expression of potential hub genes identified in the bulk expression profiles reflects the overall expression levels of genes in the whole detected tissue, it would be difficult to determine which types of cells contribute to the disorder of expression. Thus, we explored the expression levels of the potential hub genes in different cell clusters in the single-cell profile to identify CTSP hub genes. The results indicated that 23 potential hub genes were specifically expressed in macrophages, 3 genes were specifically expressed in endothelial cells, 2 in proximal tubular cells, and 1 in smooth muscle cells. Details on the CTSP hub genes are given in Supplementary Table S3.

Potential hub genes specifically expressed in macrophages included C1QA, C1QB, C3AR1, CCL4, CD14, CD36, CLEC5A, CRTAM, CSF1R, CX3CR1, CYBB, FPR3, GPR65, HCLS1, IL1B, IL10RA, LYZ, MPEG1, PLA2G7, PLEK, TGFBI, TYROBP, and VSIG4. All genes were upregulated in IgAN compared to those in normal controls in the bulk expression profile. These genes were deemed macrophage-specific potential (MSP) hub genes.

### Validation of the Differential Expression of MSP-Hub Genes in the External Dataset

DEG analysis of the MSP-hub genes in the external dataset (Ju CKD Glom from Nephroseq database) validated the differential expression of all CTSP-hub genes, except CRTAM and MPEG1. The expression of MPEG1 was not detected in the external dataset, and the logFC of CRTAM was not enough to be identified as a DEG. Details on differential expression levels of the MSP-hub genes in the external dataset are presented in Supplementary Table S4.

### Clinical Significance Analysis for MSP-Hub Genes and Identification of Real Hub Genes Specifically Expressed in Macrophage

**FIGURE 6:**
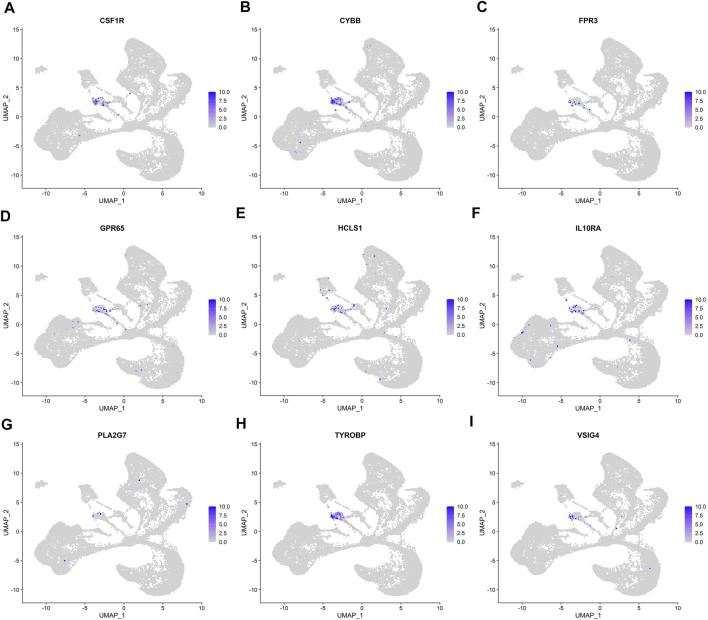
UMAP plots of the expression and distribution of nine real hub genes for each cell cluster. **(A–I)** Expression distribution of CSF1R, CYBB, FPR3, GPR65, HCLS1, IL10RA, PLA2G7, TYROBP, and VSIG4 in different cell clusters in single-cell UMAP plots.

**FIGURE 7:**
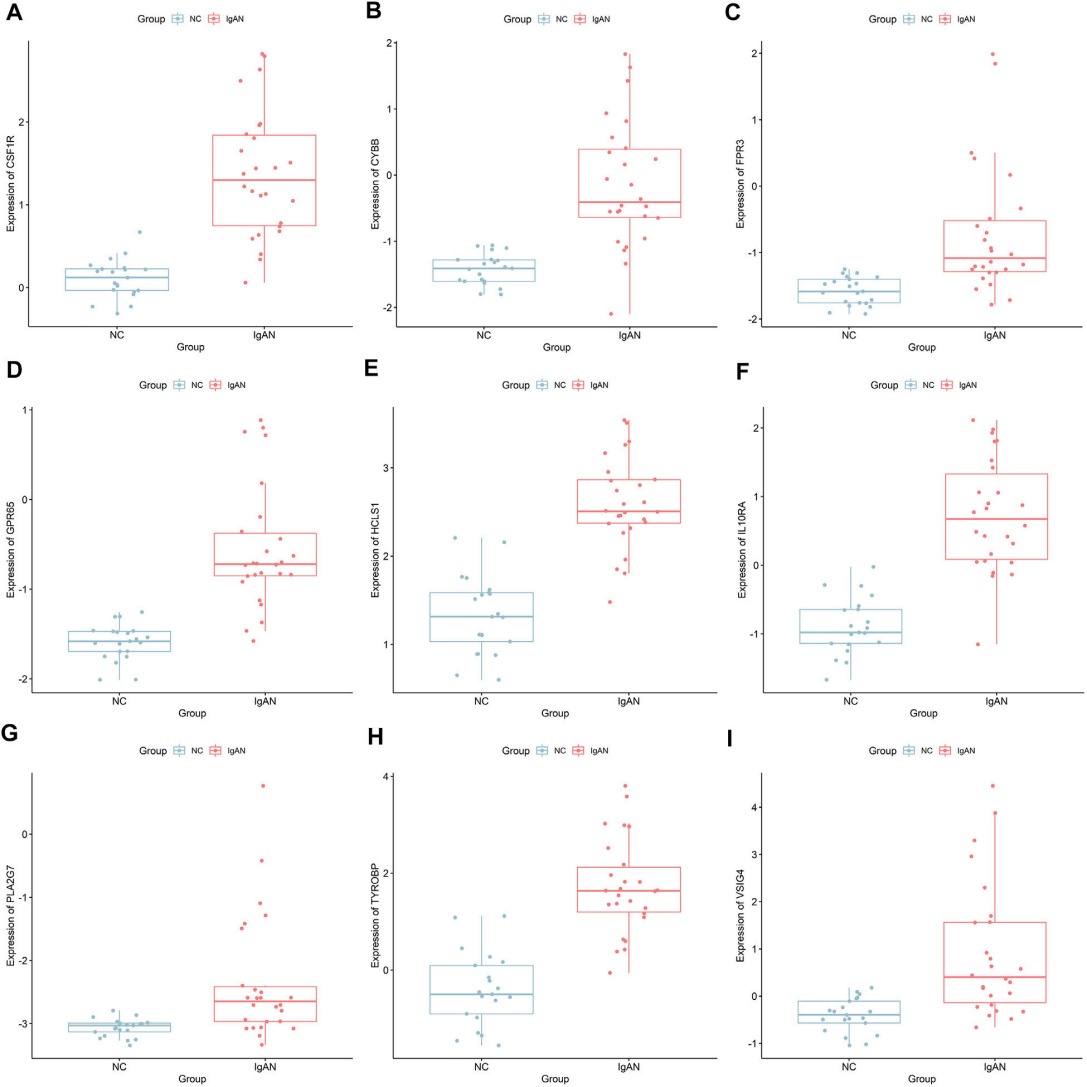
Differential expression of the nine real hub genes between IgAN and normal control in the external dataset. **(A–I)** Differential expression of CSF1R, CYBB, FPR3, GPR65, HCLS1, IL10RA, PLA2G7, TYROBP, and VSIG4 in the external dataset.

**FIGURE 8:**
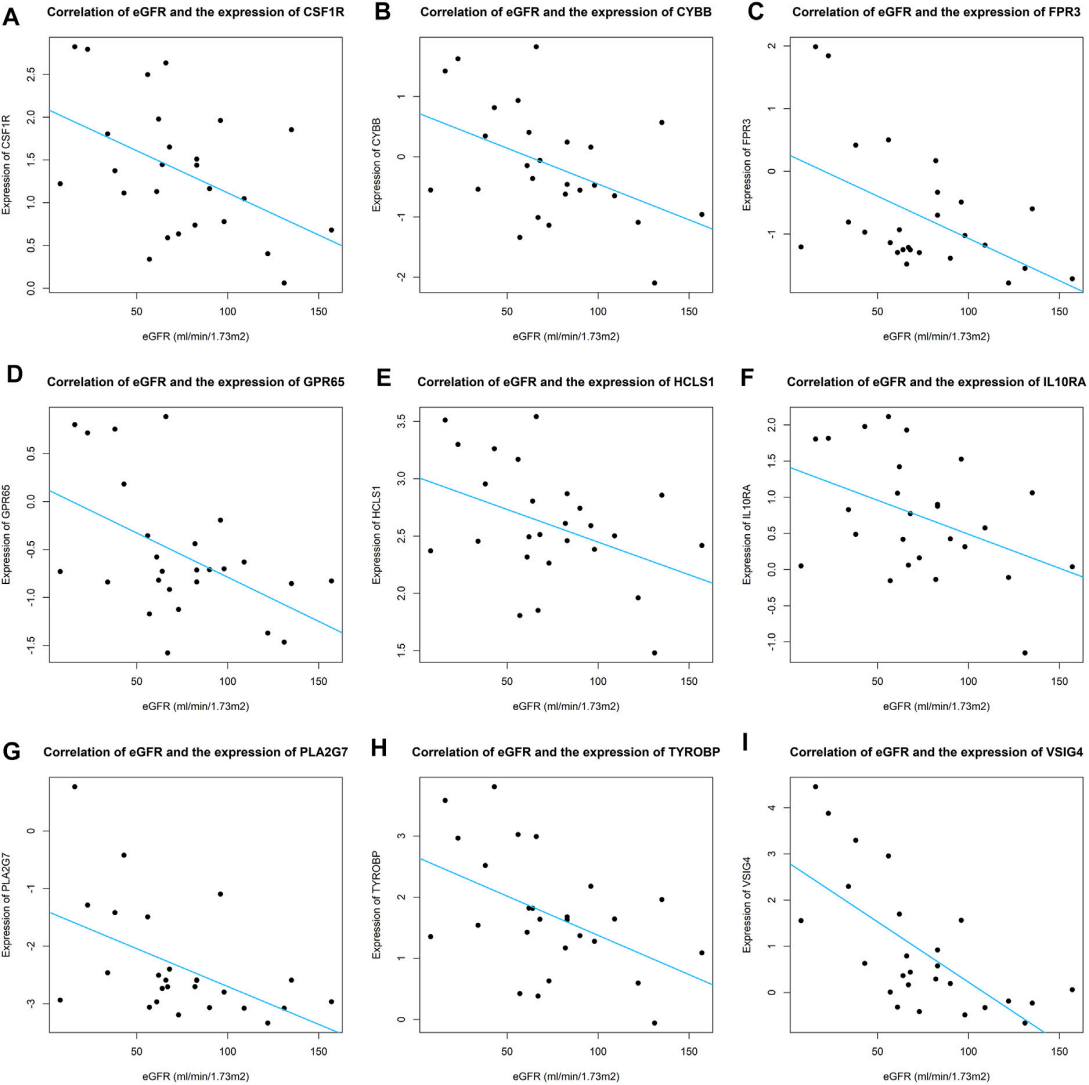
Correlation of eGFR and expression of real hub genes across IgAN patients. **(A–I)** Correlation of eGFR and expression of CSF1R, CYBB, FPR3, GPR65, HCLS1, IL10RA, PLA2G7, TYROBP, and VSIG4.

We analyzed the correlation between eGFR and the expression levels of MSP-hub genes in IgAN cases and identified genes with prognostic significance as real hub genes. The real hub genes specifically expressed in macrophages included CSF1R, CYBB, FPR3, GPR65, HCLS1, IL10RA, PLA2G7, TYROBP, and VSIG4 (Table 2). The expression levels of these nine real hub genes were significantly negatively correlated with the eGFR. The expression patterns of the nine real hub genes in different cell clusters in the single-cell profile are shown in Figure 6, Supplementary Figure S4, and Supplementary Table S3. The differential expression of the real hub genes validated in the external dataset is shown in Figure 7. The correlation between expression levels of the real hub genes and eGFR is shown in Figure 8.

**TABLE 2:** Information of the nine real hub genes.

Gene symbol	Ensemble ID	Genomic location	Correlation coefficient of gene expression and eGFR	*p*-value (E)
CSF1R	ENSG00000182578	chr5:150,053,291-150,113,372	−0.477	1.38-2
CYBB	ENSG00000165168	chrX:37,639,312-37,672,714	−0.463	1.71-2
FPR3	ENSG00000187474	chr19:51,795,157-51,826,207	−0.512	7.46-3
GPR65	ENSG00000140030	chr14:88,471,479-88,481,155	−0.495	1.02-2
HCLS1	ENSG00000180353	chr3:121,350,246-121,379,750	−0.412	3.63-2
IL10RA	ENSG00000110324	chr11:117,857,109-117,873,752	−0.427	2.94-2
PLA2G7	ENSG00000146070	chr6:46,671,938-46,703,458	−0.502	8.90-3
TYROBP	ENSG00000011600	chr19:35,904,401-35,908,295	−0.485	1.20-2
VSIG4	ENSG00000155659	chrX:66,021,738-66,040,125	−0.684	1.18-4

The nine real hub genes specifically expressed in macrophages were upregulated in IgAN compared to those in normal controls and made great biological sense in the progression of IgAN. Higher expression levels of the nine real hub genes indicated worse outcomes in patients with IgAN.

### Function Prediction of Real Hub Genes via Single-Gene GSEA

**FIGURE 9:**
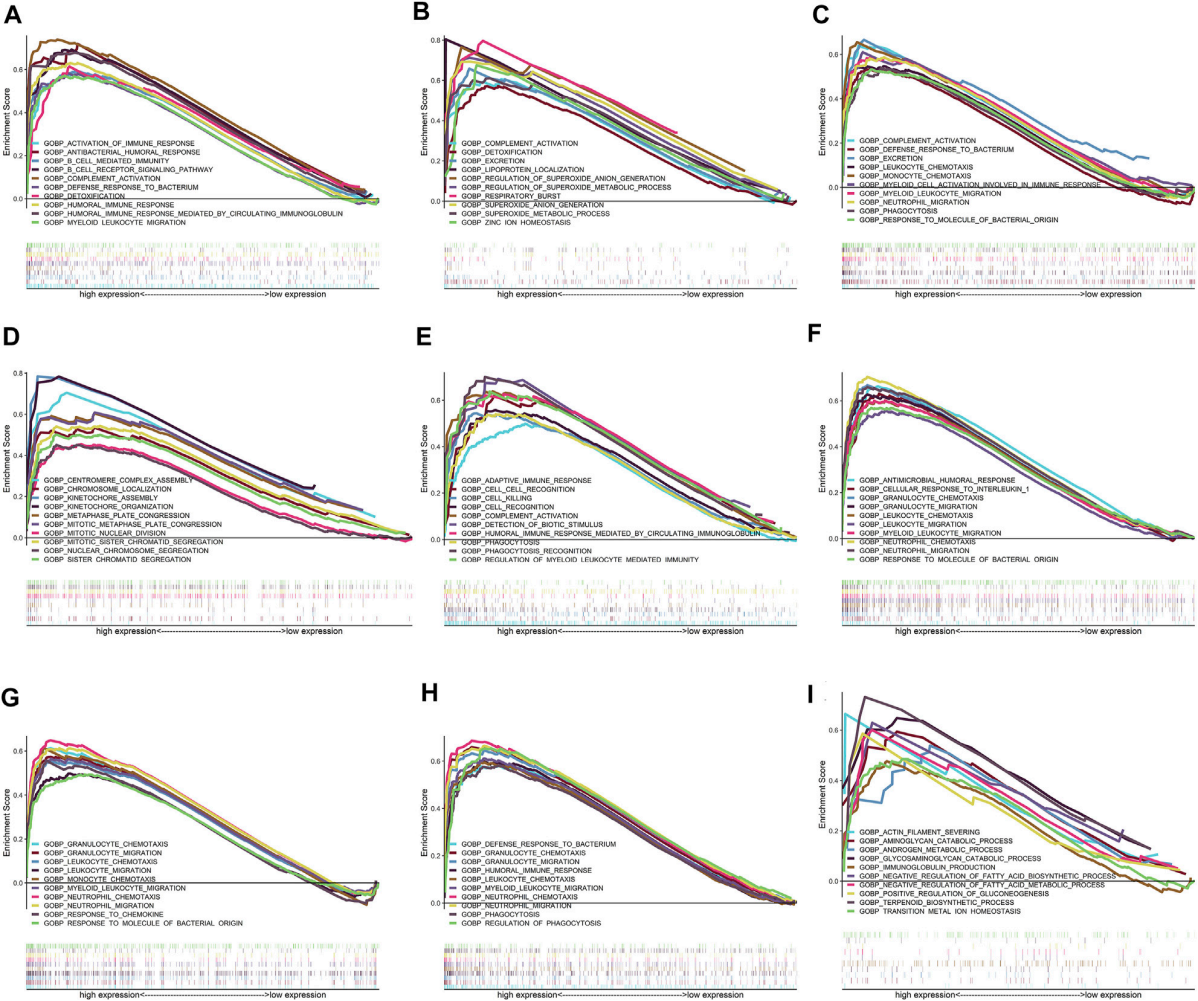
Results of GSEA for the nine real hub genes. Only top 10 upregulated terms in high-expression groups of the nine real hub genes in IgAN cases are exhibited. **(A–I)** Top 10 upregulated GO-BP terms in the high-expression group of CSF1R, CYBB, FPR3, GPR65, HCLS1, IL10RA, PLA2G7, TYROBP, and VSIG4 in GSEA.

The results of single-cell GSEA showed anomalously regulated biological processes in the high-expression groups of the real hub genes compared with those in the low-expression groups in patients with IgAN. For example, the upregulation of CSF1R was related to the activation of immunoreactions, such as “complement activation,” “activation of immune response,” and “B-cell receptor signaling pathway.” The activation of CYBB might regulate oxidative stress, such as “superoxide anion generation.” The upregulation of FRP3 was related to “neutrophil migration,” “phagocytosis,” and “monocyte chemotaxis.” The activation of GPR65 might participate in cell proliferation, such as “mitotic sister chromatid segregation” and “kinetochore organization.” HCLS1 might be involved in cell killing and phagocytosis. The upregulation of VSIG4 was related to material metabolism in IgAN, such as “positive regulation of gluconeogenesis,” “negative regulation of fatty acid biosynthetic process,” and “glycosaminoglycan catabolic process.” IL10RA, TYROBP, and PLA2G7 are thought to participate in the regulation of chemotaxis and the migration of immune cells. Figure 9 shows the top 10 upregulated biological processes in the high-expression group of the nine real hub genes in IgAN.

## Discussion

In this study, we aimed to identify novel regulators specifically expressed in immune cell clusters that influence the outcome of patients with IgAN. A total of eleven cell clusters were verified in the scRNA-seq profile, among which an increased proportion of macrophages was observed in the IgAN group compared with that in the normal control, indicating that macrophages were recruited and activated in the renal tissue of IgAN. Next, through WGCNA and GO enrichment analysis of the bulk gene expression profiles of glomerular tissue, we identified one co-expression module that was correlated with the activation of the immune response in IgAN. Furthermore, DEG analysis of genes enriched in GO terms in the IgAN-related and immune-involving module revealed 45 upregulated genes as potential hub genes. We then characterized the expression locations of these potential hub genes in single-cell clustering and revealed that 23 potential hub genes were specifically expressed in macrophages, 3 genes were specifically expressed in endothelial cells, 2 in proximal tubular cells, and 1 in smooth muscle cells. Applying the Nephroseq database, we obtained nine real hub genes that were significantly associated with the levels of eGFR in macrophages, including CSF1R, CYBB, FPR3, GPR65, HCLS1, IL10RA, PLA2G7, TYROBP, and VSIG4. To further explore the underlying mechanisms of the real hub genes specifically expressed in macrophages in IgAN, GSEA was utilized to predict the functions of these genes in IgAN. CSF1R, FRP3, HCLS1, IL10RA, PLA2G7, and TYROBP were found to contribute to the regulation of immune cells and immunoreaction. CYBB might regulate oxidative stress, GPR65 might participate in cell proliferation, and VSIG4 was involved in material metabolism.

CD45^+^ leukocyte infiltration is associated with a poor outcome of more severe mesangial cell proliferation, extracellular matrix expansion, glomerular sclerosis, and interstitial fibrosis in IgAN (Takahata et al., 2020). Moreover, studies have shown that macrophages constitute the predominant infiltrating cell types in IgAN kidneys (Wang and Harris, 2019; Xie et al., 2021). However, knowledge regarding the function of macrophages in IgAN kidneys is still lacking. Our study validated the infiltration of macrophages in the renal tissue of IgAN patients and extracted nine novel hub genes specifically expressed in macrophages and related to the clinical prognosis of IgAN patients.

The functions of the nine hub genes identified in our study have been partly clarified in macrophages and kidney disease. CSF1R encodes the receptor of colony-stimulating factor 1 (CSF1), which mediates most biological effects of CSF1, including production, differentiation, and function in macrophages (Ordentlich, 2021). CSF1 blockade reduces kidney-resident macrophages (Lin et al., 2019). One study on the progression from acute kidney injury (AKI) to chronic kidney disease (CKD) in an ischemia–reperfusion-induced AKI model showed that the inhibition of CSF1R could significantly reduce the number of macrophages infiltrating the kidney and attenuate kidney injury and interstitial fibrosis by reducing the level of Ly6C + inflammatory macrophages (Deng et al., 2020). Elevated levels of CSF1 in serum, urine, and kidneys are correlated with the activity of lupus nephritis (Menke et al., 2009), and CSF1R inhibition could deplete macrophages and ameliorate kidney injury by decreasing proteinuria and blood urea nitrogen and improving renal histopathology (Chalmers et al., 2017). CSF1R might aggravate kidney injury through similar mechanisms in IgAN. TYROBP encodes a transmembrane signaling polypeptide primarily expressed in macrophages, NK cells, dendritic cells, and neutrophils (Bakker et al., 1999). TYROBP is essential for the survival, proliferation, and function of mononuclear phagocytes (Otero et al., 2009). TREM1 and TREM2 are receptors of TYROBP, and the inflammatory response can be activated through the TREM1-TYROBP signaling pathway (Carrasco et al., 2019). The infiltration of macrophages and upregulation of TYROBP contribute to the aggravation of inflammation in obstructive nephropathy (Tammaro et al., 2013). Similarly, TYROBP promotes the survival of tissue-resident macrophages and facilitates the production of neutrophil chemoattractants in pulmonary ischemia–reperfusion injury (Spahn et al., 2015). CYBB, also known as NADPH oxidase 2 (NOX2), encodes the beta chain of cytochrome b-245 and is an important component of the oxidase system of phagocytes. CYBB is upregulated in interstitial macrophages and is involved in the pathogenesis of fibrosis in kidney allografts (Djamali et al., 2009). CYBB promotes macrophage infiltration (You et al., 2013) in diabetic kidney disease; conversely, inhibition of NOX2 attenuates podocyte injury and oxidative stress (Zhou et al., 2013). The protein encoded by IL10RA is a receptor for interleukin 10 (IL-10). IL-10 is a multifunctional immune regulator in diverse inflammatory diseases, including kidney disease (Huang et al., 2000; Diefenhardt et al., 2018). The effect of IL10RA upregulation on macrophages in glomerulopathy has not been well illustrated. One study on lung injury revealed high expression of IL10RA in macrophages and its induction of fibroblast activation after injury (Bhattacharyya et al., 2022). VSIG4, also referred to as the complement receptor of the immunoglobulin (CRIg) superfamily, encodes a protein of v-set and immunoglobulin domain-containing 4 that is exclusively expressed on tissue-resident macrophages (He et al., 2008) and is regarded as a new regulator of immunity. VSIG4 inhibited inflammation and cell death in AKI after kidney transplantation (Zhang et al., 2021) and other inflammatory diseases. Phospholipase A2 group VII, encoded by PLA2G7, is a secreted enzyme mainly expressed in macrophages and platelets. Although the functions of PLA2G7 in kidney disease have rarely been reported, it has been broadly reported that the overexpression of PLA2G7 represents the activation of macrophages and increases the inflammatory action (De Keyzer et al., 2009; Ferguson et al., 2012). FPR3 encodes N-formyl peptide receptor 3 and is expressed in various cell types, including macrophages, which are involved in innate immunity and inflammation (Schepetkin et al., 2011). FPR3 may regulate macrophage chemotaxis (Mao et al., 2021), but its function in macrophages remains unclear. GPR65 encodes G protein-coupled receptor (GPR) 65, which enables GPR activity and is involved in various biological processes. Deletion of GPR65 reduces proinflammatory M1 macrophages (Dai et al., 2020) and alters the cell pH (Li et al., 2013). However, the role of GPR65 in kidney injury has not yet been clarified. HCLS1 encodes hematopoietic cell-specific Lyn substrate 1, the function of which has not been well studied in macrophages.

The primary innovation of the present study is the integrated analysis of the bulk RNA and single-cell profiles of IgAN. Previous studies have shown the landscape of the expression pattern of IgAN at the bulk transcriptome level. However, the bulk expression profile could only reflect the overall levels of certain genes in the whole tissue but could not clarify the location of the genes in the tissue. Even if the relationship between the expression conditions of given genes and diseases is definite, it would be difficult to clarify the cell types that actually express the genes and impact the pathogenesis of diseases, which hinders further mechanistic research. Single-cell sequencing solves the problem of gene expression localization; however, the sample size for sequencing is often relatively small owing to the high price, which also limits the advantages of this method. Our study innovatively combined the bulk transcriptome profile with a single-cell profile of IgAN for the first time to identify novel hub genes that are specifically expressed in kidney-resident macrophages. Furthermore, the prognostic value of novel biological markers was verified through a combination of expression profiles and clinical data.

Nonetheless, our study has some limitations. First, the sample size of the single-cell dataset was relatively small. Our findings should be further validated in a larger dataset, if available. Second, the detailed functions of the nine novel hub genes in IgAN have not been clarified, and further experimental and clinical studies are needed.

## Conclusion

We conducted comprehensive bioinformatics analyses by integrating single-cell dataset, bulk dataset, and clinical data of IgAN for the first time. The results highlighted the significance of macrophage infiltration in glomeruli in IgAN and identified nine novel hub genes specifically expressed in macrophages that could act as novel prognostic biomarkers for IgAN patients. These conclusions may help interpret the molecular mechanisms underlying IgAN and provide new therapeutic strategies.

## Data Availability

The original contributions presented in the study are included in the article/Supplementary Material, further inquiries can be directed to the corresponding author.

## Supplementary Material

The Supplementary Material for this article can be found online at: https://www.frontiersin.org/articles/10.3389/fmolb.2022.884588/full#supplementary-material

Supplementary Figure S1 ElbowPlot analysis utilized for selected principal components. Fifteen principal components were selected for UMAP drafting.

Supplementary Figure S2 Cluster dendrogram with clinical trait heatmap of IgAN and normal control sample based on their expression profiles.

Supplementary Figure S3 Determination of the proper threshold of the *β*-value in WGCNA.
**(A)**Analysis of the scale-free fit index of each *β*-value from 1 to 20. **(B)**Analysis of average connectivity of each *β*-value from 1 to 20. *β* = 7 was chosen for subsequent analyses, as it has the largest average connectivity when the scale-free fit index is up to 0.85.

Supplementary Figure S4 Dot plots of the expression levels of the nine real hub genes across 11 cell clusters. The size of the dot represents the proportion of the cell population that expresses the gene. Shading of color indicates the expression level of the gene.
