# Supplementary figures and images for: Identification and Validation of Prognostic Biomarkers Specifically Expressed in Macrophage in IgA Nephropathy Patients Based on Integrated Bioinformatics Analyses
Source: Front Mol Biosci. 2022 May 5;9:884588. doi: 10.3389/fmolb.2022.884588 (PMC9117719; doi:10.3389/fmolb.2022.884588)

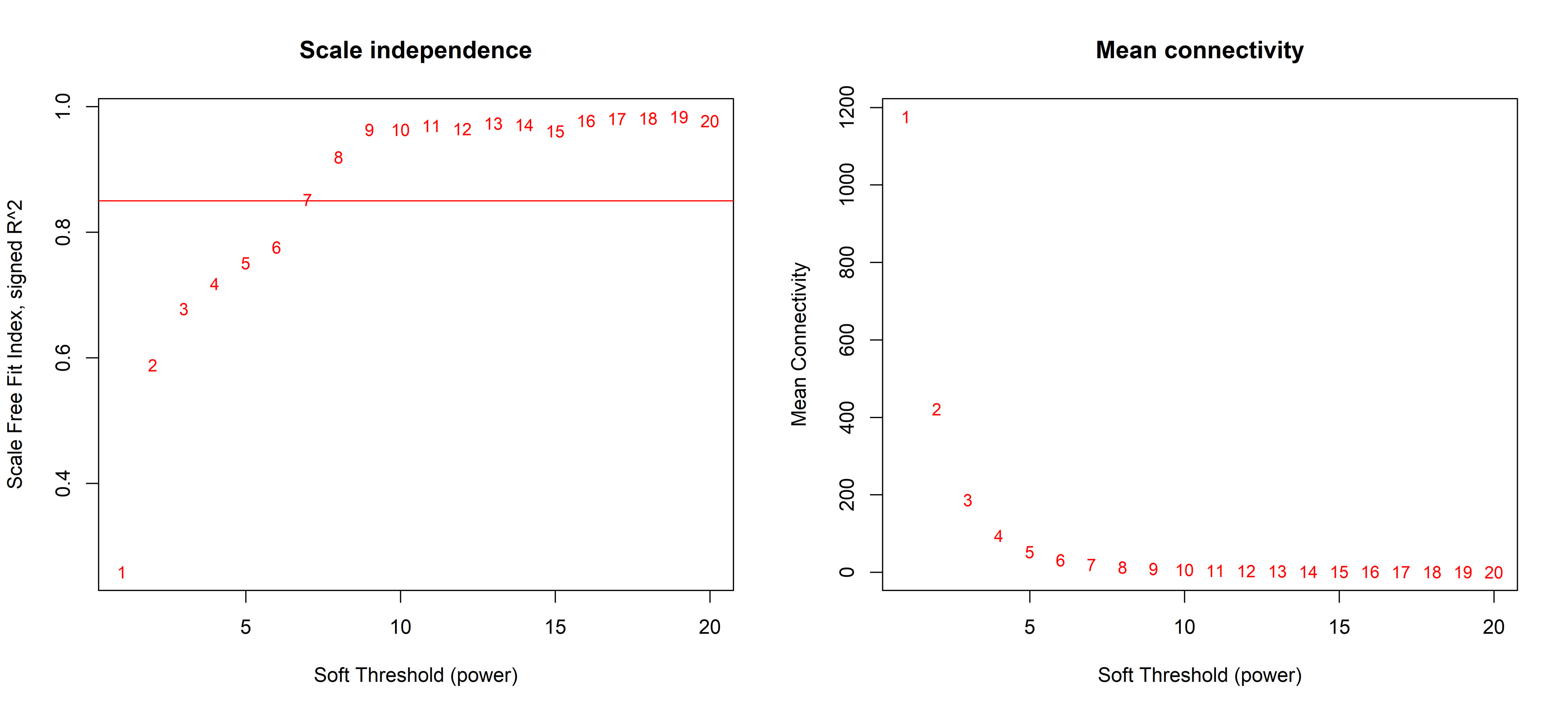

Supplement: Supplementary file 2 [file Image3.JPEG]

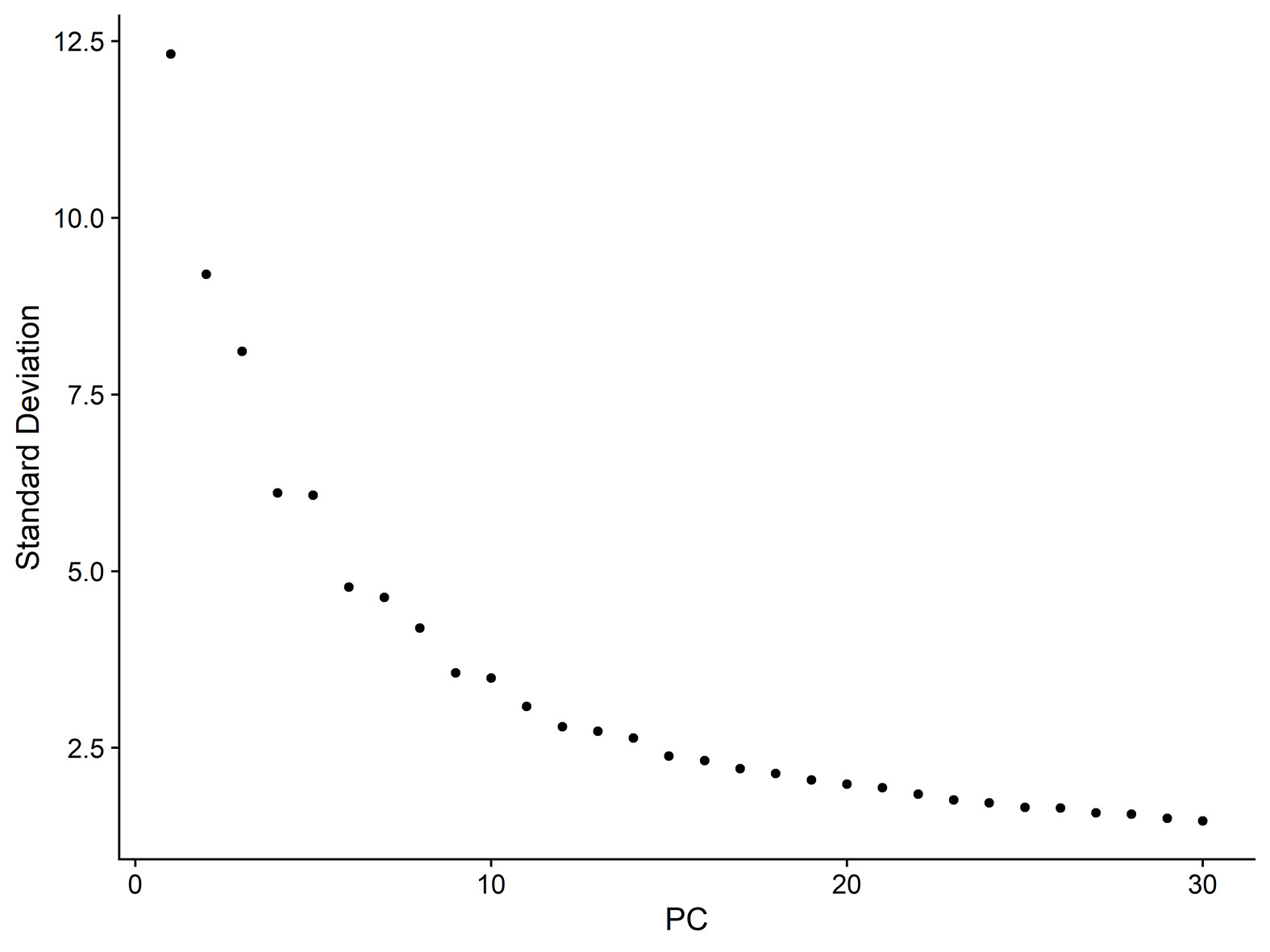

Supplement: Supplementary file 4 [file Image1.JPEG]

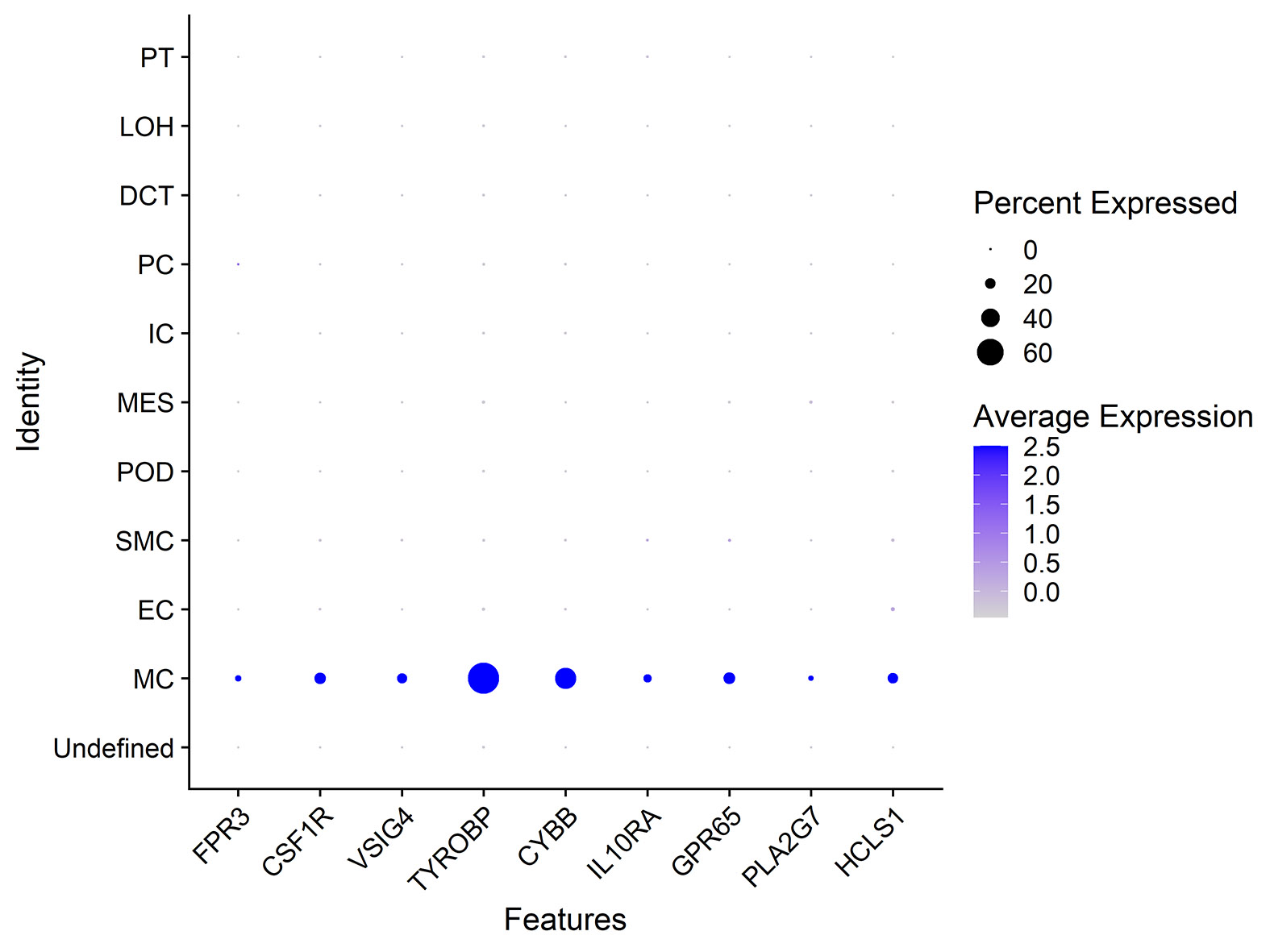

Supplement: Supplementary file 5 [file Image4.JPEG]

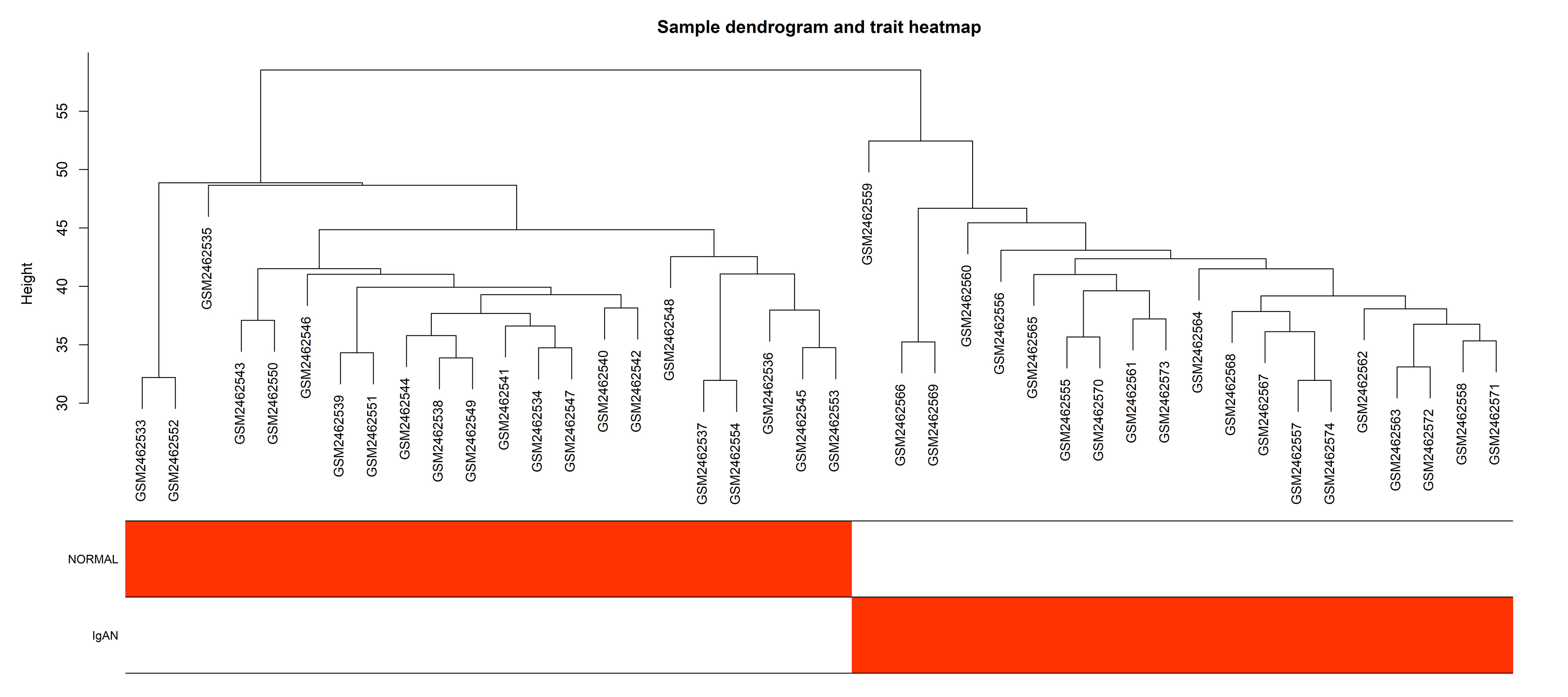

Supplement: Supplementary file 6 [file Image2.JPEG]
